# Enhanced thermoelectric properties of Zn-doped GaSb nanocomposites

**DOI:** 10.1039/d0ra00898b

**Published:** 2020-07-30

**Authors:** Qiang Fu, Zhimin Wu, Jiapeng Li

**Affiliations:** School of Mechanical and Electrical Engineering, Shenzhen Polytechnic Shenzhen 518005 People's Republic of China fuqiang@szpt.edu.cn; Department of Mechanical and Automation Engineering, The Chinese University of Hong Kong Shatin New Territories Hong Kong Special Administration Region People's Republic of China; School of Mechanical Engineering, Shandong University of Technology Zibo 255049 People's Republic of China

## Abstract

In this work, Zn-doped GaSb nanocomposites (Ga_1−*x*_Zn_*x*_Sb, *x* = 0.002, 0.005, 0.01, and 0.015) have been synthesized *via* ball milling followed by hot pressing. It is shown that thermoelectric properties of the synthesized Ga_1−*x*_Zn_*x*_Sb nanocomposites vary with both the grain size and the Zn content. The grain boundaries formed in the nanocomposites not only scatter phonons and reduce thermal conductivity, but also trap charge carriers and reduce electrical conductivity. Zn doping is adopted to compensate for the trapping effect of grain boundaries on carrier transport in order to enhance the thermoelectric figure of merit, *ZT*, of Ga_1−*x*_Zn_*x*_Sb alloys. By optimizing the amount of Zn doping, the maximum *ZT* value was found to be 0.087 at 500 K for Ga_0.99_Zn_0.01_Sb nanocomposites, which is 51% higher than the reported value in the literature for bulk Ga_1−*x*_Zn_*x*_Sb alloys.

## Abstract 2

The maximum *ZT* value of Zn-doped GaSb nanocomposites was improved by 51% corresponding to the literature bulk value.

## Graphical abstract

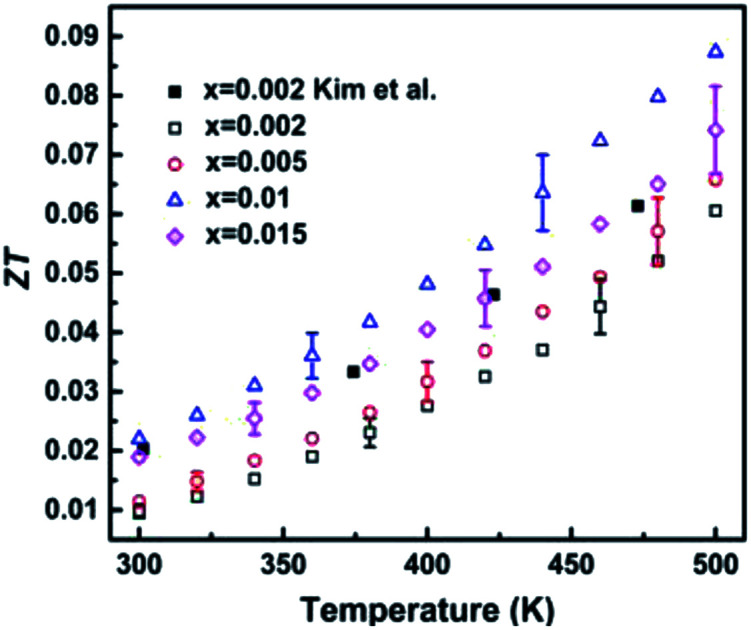

## 1. Introduction

With the increasing energy demand and growing environmental problems caused by burning fossil fuels, thermoelectric materials have drawn much attention in the past few decades for their potential in converting waste heat to electrical power. The energy conversion efficiency of thermoelectric materials is determined by a dimensionless figure of merit, *ZT* = *S*^2^*σT*/*κ*, where *S* is the Seebeck coefficient, *σ* is the electrical conductivity, *κ* is the thermal conductivity, and *T* is the absolute temperature. To achieve high *ZT* values, a high power factor (*S*^2^*σ*) and low thermal conductivity (*κ*) are desired. However, it is difficult to tune these properties independently in conventional bulk materials since they are coupled to each other.

In the past two decades, research on thermoelectric materials has mainly focused on utilizing nanostructures to improve *ZT* values. A large amount of grain boundaries commonly exist in nanostructured materials, which can effectively scatter phonons with different wavelengths and thus reduce the lattice thermal conductivity. Two approaches have been widely used to synthesize various thermoelectric nanocomposites. One approach is to incorporate nanoinclusions into bulk or thin film materials, as demonstrated by Ag_1−*x*_Pb_18_SbTe_20_ (LAST) with nanoscale inhomogeneities embedded in the PbTe matrix,^1^ In_0.47_Ga_0.53_As alloys with embedded ErAs nanoparticles,^2^ and In_1−*x*_Ga_*x*_Sb system with precipitated Ga-poor nanoinclusions.^3^ The other approach is to form bulk nanograined materials from nanopowders by hot pressing, such as Bi_2_Te_3_-based nanocomposites,^4,5^ SiGe nanocomposites,^6,7^ and PbTe-based nanocomposites.^8^ In addition to the reduced lattice thermal conductivity, the Seebeck coefficient of nanostructured materials can be enhanced by increasing density of states of electrons^9,10^ or filtering of low-energy carriers by the potential barrier at interfaces.^11,12^ However, grain boundaries in nanostructured materials not only affect phonon transport but also have a trapping effect on charge carriers.^13–15^ In some instances, the *ZT* value cannot be improved because the benefit of the suppressed lattice thermal conductivity is offset by the deterioration of electrical conductivity.^16^ Although the trapping effect can be weakened by increasing the doping concentration to reduce the potential barrier height at grain boundaries,^17,18^ the high doping concentration may also reduce the Seebeck coefficient. Thus, it is essential to tune the doping concentration to optimize the *ZT* value for nanostructured materials.

This work is focused on the Zn-doped GaSb system. GaSb is an III–V compound semiconductor with a zinc blende structure, which demonstrates a high Seebeck coefficient of about 700 μV K^−1^ (ref. 19) but also a high thermal conductivity of ∼25 W m-K^−1^ at 300 K.^20^ The high thermal conductivity of GaSb limits its thermoelectric performance. In order to enhance its *ZT* value, we synthesized Ga_1−*x*_Zn_*x*_Sb nanocomposites by ball milling and hot pressing to reduce the thermal conductivity and tuned the Zn content to optimize the power factor. The sample with *x* = 0.01 exhibited the highest *ZT* value of 0.087 at 500 K, which is 51% higher than the reported value in the literature for bulk Ga_1−*x*_Zn_*x*_Sb alloys.^20^

## 2. Experimental methodology

### 2.1. Synthesis and characterization of Ga_1−*x*_Zn_*x*_Sb nanocomposites

The starting materials, Ga_1−*x*_Zn_*x*_Sb granules (*x* = 0.002, 0.005, 0.01, and 0.015), were purchased from Jiangxi Ketai Advanced Materials Co., Ltd. The purchased granules were first ground into nanopowders by using a planetary micro mill (Pulverisette 7, Fritsch) with zirconium oxide grinding jars and balls. The grinding balls were 10 mm in diameter and the weight ratio of the balls to the powder was 15 : 1. In each run, the total amount of powder mixture used was about 5.0 g. The ball milled powders were loaded into a graphite die and sintered into a thin pellet with a diameter of 15 mm by using a DC-current-induced hot pressing system. The sintering temperature, pressure, and time were set as 650 °C, 50 MPa, and 5 min in this work in order to obtain fully dense nanograined samples. The hot pressing process was conducted in a glove box filled with argon gas to prevent the samples from oxidation caused by oxygen and water vapor in the ambient environment. The morphology of the hot-pressed samples was examined by using scanning electron microscopy (SEM, JSM-7800F, JEOL). The crystalline structure of the samples was characterized by X-ray diffraction (XRD, SmartLab diffractometer, Rigaku) using Cu K_α_ radiation at room temperature in air. The Hall coefficients at room temperature were measured using a physical properties measurement system under a reversible magnetic field up to 1.5 T (PPMS, Model 6000, Quantum Design).

### 2.2. Thermoelectric properties characterization

The hot-pressed pellet was cut and polished into a thin bar and a small disk for the characterization of thermoelectric properties. The thin-bar-shaped sample was used to measure electrical conductivity by a four-probe method and Seebeck coefficient by a quasi-steady-state method. A customized setup is used for characterizing electrical conductivity and Seebeck coefficient and experimental details can be found in our previous publication.^21^ The disk-shaped sample is used to measure thermal conductivity by a three-omega (3*ω*) method.^22^ The 3*ω* method is a commonly used technique to measure thermal conductivity of bulk materials^22^ and thin films.^23,24^ For the 3*ω* measurement, the hot-pressed sample was first polished to give the surface a fine finish. Then, 2 μm thick SiO_2_ was deposited on top of the sample surface as an insulating layer by using plasma enhanced chemical vapor deposition (PECVD). Next, a thin Au heater line with four probes was fabricated on top of the insulating layer *via* a lift-off process. The width of the heater line is 30 μm and the distance between two inner probes (the heater length) is 1 mm. During a measurement, an AC current with a frequency of *ω* was passed through two outer probes. The voltage drop between two inner probes at a frequency of 3*ω* was measured, which is related to the surface temperature rise at a frequency of 2*ω* (Δ*T*_2*ω*_). At each temperature, the measurement was conducted in a certain frequency range. Thermal conductivity of the nanocomposite sample can be extracted from the slope of the Δ*T*_2*ω*_*vs.* ln(*ω*) curve. The details of the 3ω method can be found in the literature.^22^ Thermoelectric properties measurements were conducted in a cryostat under a high vacuum with a pressure lower than 10^−6^ torr to minimize the heat loss. The measurements were performed in a temperature range from 300 K to 500 K.

## 3. Results and discussion

### 3.1. Effect of ball milling conditions

We first studied the effect of ball milling conditions on the morphology and thermoelectric properties of Ga_1−*x*_Zn_*x*_Sb nanocomposites. In this part, the Zn content *x* is fixed at 0.005. A set of Ga_0.995_Zn_0.005_Sb nanocomposites was synthesized using powders prepared at different combinations of ball milling speeds and times including 600 rpm–3 h, 1000 rpm–3 h, and 1000 rpm–6 h. The hot pressing conditions are the same for all the samples as mentioned above.

**Fig. 1:**
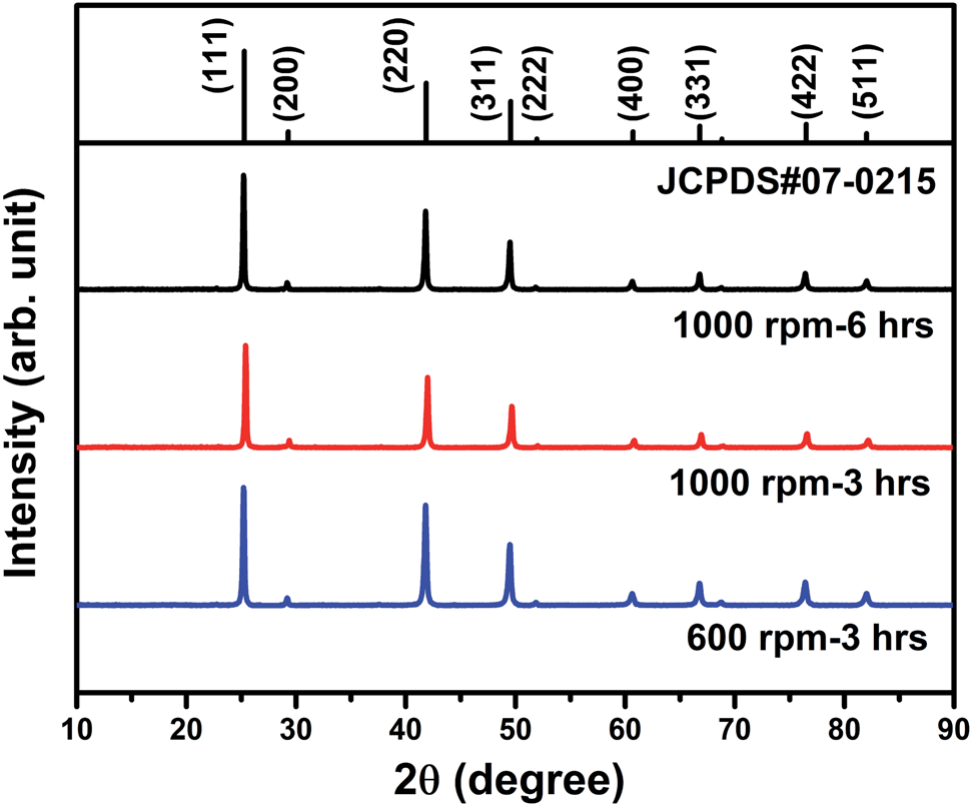
XRD patterns of the hot-pressed Ga_0.995_Zn_0.005_Sb nanocomposites using powders prepared at different ball milling conditions.

**Fig. 2:**
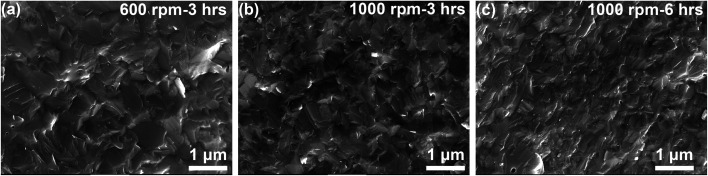
SEM images of the hot-pressed Ga_0.995_Zn_0.005_Sb nanocomposites using powders prepared at different ball milling conditions.

Fig. 1 shows the XRD patterns of the hot-pressed Ga_0.995_Zn_0.005_Sb nanocomposites. All the peaks correspond to the zinc blende structure and no secondary phase peak is observed, which confirms that all the samples are single phase in the solid-solution form. The microstructure of three Ga_0.995_Zn_0.005_Sb samples was examined by using SEM and the images are shown in Fig. 2. The SEM image was taken on the freshly broken surface for each sample. It is clearly shown that the sample prepared at higher ball milling speed and longer time demonstrates smaller grain sizes.

**Fig. 3:**
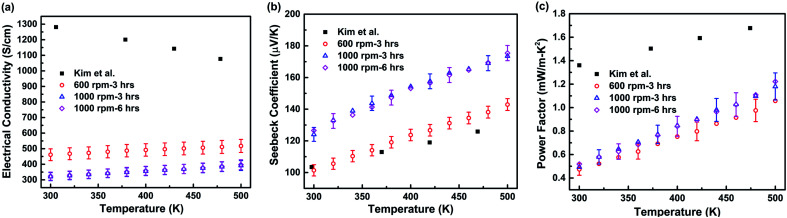
Temperature-dependent electrical properties of the hot-pressed Ga_0.995_Zn_0.005_Sb nanocomposites using powders prepared at different ball milling conditions. (a) Electrical conductivity; (b) Seebeck coefficient; (c) power factor. The uncertainties in electrical conductivity, Seebeck coefficient, and power factor are estimated to be less than 8%, 4%, and 12%, respectively. Detailed uncertainties analysis for electrical conductivity and Seebeck coefficient measurement can be found in ref. 21. The results reported for the bulk alloy by Kim *et al.*^20^ are also plotted for comparison.

Fig. 3 shows electrical properties of the hot-pressed Ga_0.995_Zn_0.005_Sb nanocomposites in the temperature range from 300 K to 500 K. The data for the bulk Ga_0.995_Zn_0.005_Sb alloy from Kim *et al.*^20^ is also plotted for comparison. As seen in Fig. 3(a), the electrical conductivity of all the Ga_0.995_Zn_0.005_Sb nanocomposites is much lower than the counterpart for the bulk alloy in the literature.^20^ For instance, at 300 K, the electrical conductivity of the bulk Ga_0.995_Zn_0.005_Sb alloy is about 1280 S cm^−1^, however, it is only 461 S cm^−1^ for our nanocomposite sample prepared at 600 rpm–3 h, about one third of the bulk value. The values are even lower for samples prepared at 1000 rpm–3 h and 1000 rpm–6 h. This is due to the trapping states formed at grain boundaries which create potential barrier and impede the conduction of carriers between the grains. With smaller grain size, the surface area/grain volume ratio is higher, and the potential barrier scattering effect is more significant. Therefore, when the material composition is fixed at Ga_0.995_Zn_0.005_Sb, smaller grain size would lead to lower electrical conductivity. For the bulk Ga_0.995_Zn_0.005_Sb alloy, as replotted in Fig. 3(a), *σ* decreases with the increase of temperature from 300 K to 500 K, which is the signature of a degenerate semiconductor.^20^ In our nanocomposite samples, both *σ* and *S* increases with temperature from 300 K to 500 K. This can be explained by the filtering effect of the potential energy barrier on charge carriers. In nanograined materials, potential barriers of height *V*_B_ are formed at grain boundaries, when carriers with an energy distribution pass through a potential barrier, carriers with energy lower than *V*_B_ are strongly scattered or stopped, while the carriers with higher energy can pass through the barrier. As a result, the potential barrier formed at grain boundaries filters low-energy charge carriers, which in turn enhances the Seebeck coefficient of nanograined samples.^26,27^ Therefore, the Seebeck coefficient is higher for samples with smaller grain sizes. The power factors of all Ga_0.995_Zn_0.005_Sb nanocomposites are lower than the counterpart of the bulk alloy in the literature^20^ due to the significantly reduced electrical conductivity. The power factors of nanocomposite samples prepared at 1000 rpm–3 h and 1000 rpm–6 h are higher than that of the sample prepared at 600 rpm–3 h due to the enhanced Seebeck coefficient. For both *σ* and *S*, the values for samples prepared at 1000 rpm–3 h and 1000 rpm–6 h are very close, which can be attributed to similar grain sizes of these two samples.

**Fig. 4:**
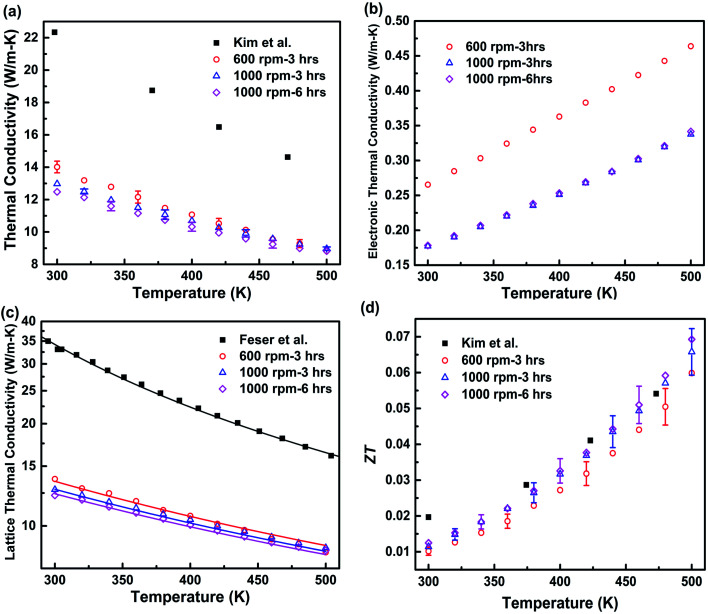
Temperature-dependent thermal conductivity and *ZT* of the hot-pressed Ga_0.995_Zn_0.005_Sb nanocomposites using powders prepared at different ball milling conditions. (a) Measured total thermal conductivity; thermal conductivity of the bulk Ga_0.995_Zn_0.005_Sb alloy reported by Kim *et al.*^20^ is also shown for comparison. (b) Electronic thermal conductivity; (c) lattice thermal conductivity; Also shown is the thermal conductivity of bulk single crystal GaSb reported by Feser *et al.*^33^ Solid lines show the simulated lattice thermal conductivity by using the Callaway model.^29^ (d) *ZT*. The uncertainties in thermal conductivity and *ZT* are estimated to be within 3% and 11%, respectively. Detailed uncertainties analysis for thermal conductivity measurement can be found in ref. 35.

Compared to the bulk data,^20^ the thermal conductivity of the hot-pressed nanocomposite samples is reduced by a factor of 1.6–1.8 at room temperature, as shown in Fig. 4(a), a clear sign of the enhanced phonon grain boundary scattering effect. According to the Wiedemann–Franz law, the electronic thermal conductivity can be estimated by *κ*_e_ = *LσT*, where the Lorentz number *L* can be determined by using the following equation proposed by Kim *et al.*,^28^1
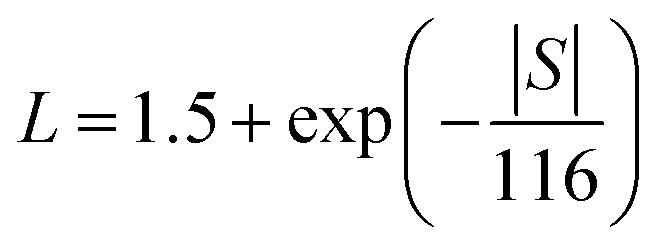
where *S* is in μV K^−1^. The lattice thermal conductivity can be calculated by subtracting the electronic contribution from the total thermal conductivity, *κ*_l_ = *κ* − *κ*_e_. The calculated *κ*_e_ and *κ*_l_ are shown in Fig. 4(b) and (c), respectively. The *κ*_l_ of all the nanocomposites is very close to *κ*, suggesting that the lattice contribution is dominant in the total thermal conductivity for the hot-pressed Ga_0.995_Zn_0.005_Sb nanocomposites. The *κ*_l_ shows a decreasing trend with temperature in the measured temperature range, revealing that Umklapp scattering plays a significant role in phonon transport. The temperature dependence of *κ*_l_ is simulated by using the Callaway model that is based on the Boltzmann transport equation with the relaxation time approximation:^29^2
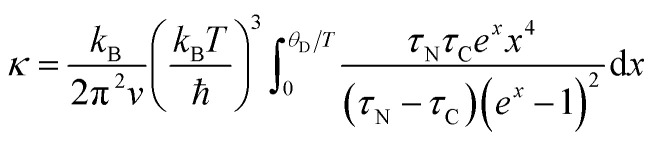
where *v* is the acoustic phonon group velocity, ℏ is the reduced Plank constant, *θ*_D_ is the Debye temperature, and *x* = ℏ*ω*/*k*_B_*T* is the normalized frequency. *τ*_N_ is the relaxation time due to normal scattering. *τ*_C_ is the combined relaxation time determined by using the Matthiessen's rule,^30^

3 *τ*_C_^−1^ = *τ*_U_^−1^ + *τ*_N_^−1^ + *τ*_A_^−1^ + *τ*_B_^−1^.

The contributions of Umklapp scattering (*τ*_U_), normal scattering (*τ*_N_), alloy scattering (*τ*_A_), and boundary scattering (*τ*_B_) are considered in *τ*_C_. The commonly used formulas are taken for Umklapp scattering and normal scattering:^31^*τ*_U_^−1^ = *B*_1_*ω*^2^*T*e^(−*B*_2_ /*T*)^ and *τ*_N_^−1^ = *B*_3_*ω*^2^*T*^3^. The relaxation time due to alloy scattering is calculated by *τ*_A_^−1^ = *Aω*^4^.^32^*B*_1_, *B*_2_, *B*_3_, and *A* are fitting parameters, which can be determined by fitting the thermal conductivity of bulk single crystal GaSb reported by Feser *et al.*,^33^ as shown in Fig. 4(c). The frequency-dependent model proposed by Wang *et al.*^34^ is adopted for grain boundary scattering,4
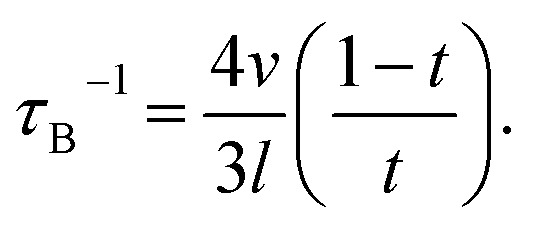
Here 
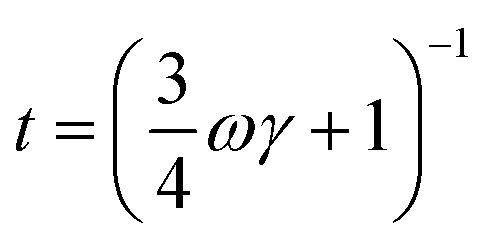
, where *γ* is a fitting parameter. Fig. 4(c) shows the modeled lattice thermal conductivity of the hot-pressed Ga_0.995_Zn_0.005_Sb nanocomposites. By adjusting *l* from 190 nm, 170 nm, to 160 nm for samples prepared at 600 rpm–3 h, 1000 rpm–3 h, and 1000 rpm–6 h, the modeled results capture the trend of the experimental data, indicating that the *κ*_l_ of the nanocomposites is depressed due to enhanced phonon grain boundary scattering and this effect is more significant for smaller grain sizes. Compared to Kim *et al.*'s work,^20^ the overall *ZT* of our Ga_0.995_Zn_0.005_Sb nanocomposites is not enhanced, as shown in Fig. 4(d), since the beneficial effect of the reduced *κ* is offset by the reduction in *σ*. The *ZT* of the sample prepared at 600 rpm–3 h is slightly lower than the values of other two hot-pressed samples, which is due to the lower power factor and higher thermal conductivity.

### 3.2. Effect of Zn doping

In general, to enhance the *ZT* of nanocomposites, the reduction in electrical conductivity should be less severe compared to the reduction in thermal conductivity. For polycrystalline materials, the electrical conductivity can be expressed as^25^5
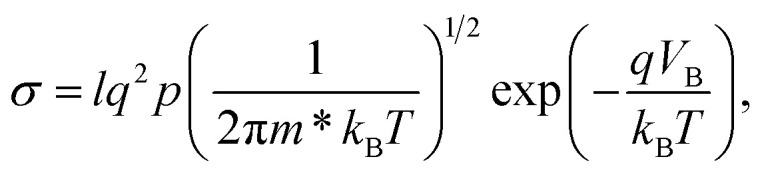
where *l* is the grain size, *q* is the elementary charge, *p* is the average carrier concentration, m* is the effective mass of charge carriers, *k*_B_ is the Boltzmann constant, and *V*_B_ is the potential barrier height. As indicated by eqn (5), the *σ* can be increased by decreasing the potential barrier height *V*_B_, which is dependent on the doping concentration. (i) When the doping concentration is low, the traps at grain boundaries are partially filled. The *V*_B_ will increase with increasing the doping concentration because the charges of the dipole layer increase as more carriers are trapped. (ii) After the traps are fully filled, with the further increase of the doping concentration, the activation energy and the potential barrier will decrease as the charges of the dipole layer remains unchanged but the width of the dipole layer is decreased. (iii) When the doping concentration is very high, the potential barrier scattering effect is eliminated and the grain boundaries do not obstruct the conduction of carriers.^18,25^ Therefore, the reduction in electrical conductivity caused by potential barrier scattering effect of grain boundaries can be weakened by high doping concentration.

**Fig. 5:**
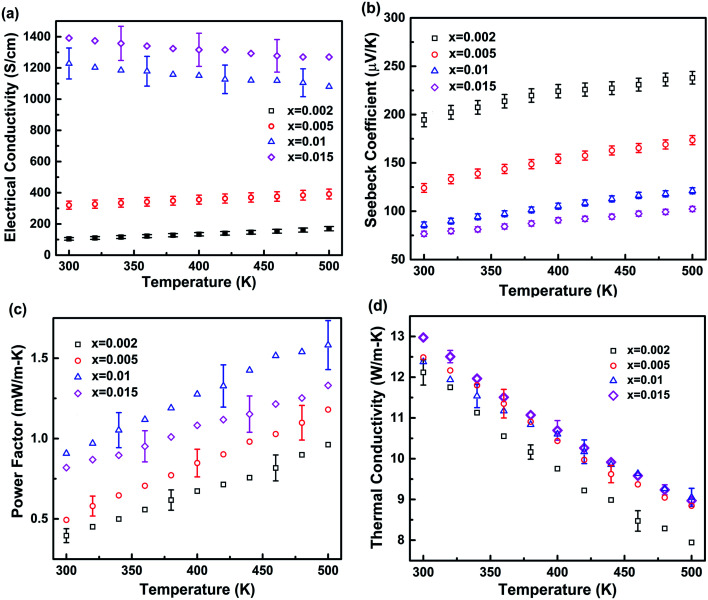
Temperature-dependent thermoelectric properties of the hot-pressed Ga_1−*x*_Zn_*x*_Sb nanocomposites with different amounts of Zn doping (*x* = 0.002, 0.005, 0.01, and 0.015). (a) Electrical conductivity; (b) Seebeck coefficient; (c) power factor; (d) thermal conductivity. The uncertainties in electrical conductivity, Seebeck coefficient, power factor, and thermal conductivity are estimated to be within 8%, 4%, 11%, and 3%, respectively.

In this study, in order to optimize the effect of grain boundaries on electrical and thermal properties and enhance the *ZT*, we synthesized a set of hot-pressed Ga_1−*x*_Zn_*x*_Sb nanocomposites with different amounts of Zn doping (*x* = 0.002, 0.005, 0.01, and 0.015). The ball milling condition is fixed at 1000 rpm–3 h. Fig. 5 shows thermoelectric properties of Ga_1−*x*_Zn_*x*_Sb nanocomposites with different amounts of Zn doping in the temperature range of 300–500 K and Table 1 lists the electrical conductivity, carrier concentration, Hall mobility and Seebeck coefficient at 300 K. The carrier concentration *p* and the Hall carrier mobility *μ* were determined from *p* = 1/(*R*_H_*q*) and *μ* = *σR*_H_, where *R*_H_ is the Hall coefficient. The *p* increases with increasing Zn content, which is consistent with the *σ* and *S* characteristics. When Zn content increased from *x* = 0.002 to 0.01, the *μ* increased from 24.1 cm^2^ V^−1^ s^−1^ to 64.0 cm^2^ V^−1^ s^−1^, we consider it belongs to (ii), the *V*_B_ decreases with increasing *p*. When Zn content increased from *x* = 0.01 to 0.015, the *μ* decreased from 64.0 cm^2^ V^−1^ s^−1^ to 48.6 cm^2^ V^−1^ s^−1^, which belongs to (iii), the *V*_B_ will not further decrease with increasing *p*, and the potential barrier scattering effect is eliminated. With the higher Zn content, the electrical conductivity is higher due to the increased carrier concentration. For *x* = 0.002 and 0.005 samples, the electrical conductivity increases with temperature, while for *x* = 0.01 and 0.015 samples, the electrical conductivity decreases with temperature. When the carrier concentration is low, the trapping effect of grain boundaries on charge carriers is significant. With the increase of temperature, charge carriers obtain more energy, which in turn helps them to overcome the potential barrier and transport across grain boundaries. When the carrier concentration is high enough, the trapping effect is eliminated and the electrical conductivity shows the signature of degenerate semiconductors. The Seebeck coefficient decreases with increasing the Zn content due to the increased carrier concentration. With the combined effects of Zn doping on both electrical conductivity and Seebeck coefficient, the Ga_0.99_Zn_0.01_Sb nanocomposite shows the highest power factor, which reaches 1.58 mW m-K^−2^ at 500 K. While for the Ga_0.985_Zn_0.015_Sb nanocomposite, although the *σ* is increased, the power factor is not further enhanced due to the reduction in *S*. For thermal conductivity, according to the Wiedemann–Franz law, the electronic contribution is marginal. Therefore, it is not surprising that thermal conductivity does not vary significantly with the Zn content. The *κ* slightly increases from 12.1 to 13.0 W m-K^−1^ at 300 K when the Zn content is increased from 0.002 to 0.015.

**Table 1:** Composition, electrical conductivity, carrier concentration, Hall mobility, Seebeck coefficient and thermal conductivity of hot-pressed Ga_1−*x*_Zn_*x*_Sb nanocomposites at 300 K

Ga_1−*x*_Zn_*x*_Sb	*σ* (S cm^−1^)	*p* (10^19^ cm^−3^)	*μ* (cm^2^ V^−1^ s^−1^)	*S* (μV K^−1^)	*κ* (W m-K^−1^)
*x* = 0.002	104	2.7	24.1	194	12.1
*x* = 0.005	320	6.7	29.9	124	12.4
*x* = 0.01	1228	12.0	64.0	86	12.5
*x* = 0.015	1391	17.9	48.6	75	13.0

**Fig. 6:**
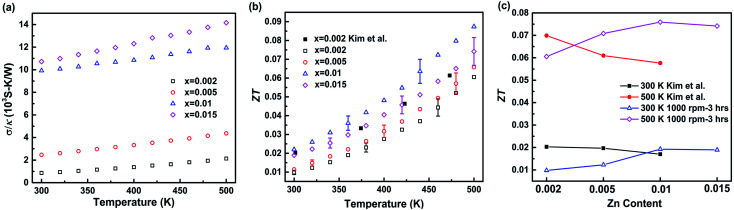
(a) Ratio of electrical conductivity to thermal conductivity and (b) *ZT* of the hot-pressed Ga_1−*x*_Zn_*x*_Sb nanocomposites with different amounts of Zn doping (*x* = 0.002, 0.005, 0.01, and 0.015); (c) variation of the *ZT* with the Zn content at 300 K and 500 K. The uncertainty in *ZT* is within 11%. The results reported by Kim *et al.*^20^ for the bulk alloy are also shown in (b) and (c) for comparison.

The grain boundaries inside nanocomposites simultaneously affect the carrier and phonon transport. They not only scatter phonons, but also scatter charge carriers. To investigate the overall effect of grain boundaries on the transport properties, the ratio of electrical conductivity to thermal conductivity is calculated and shown in Fig. 6(a). It is shown that with higher Zn content or higher temperature, the *σ*/*κ* is larger, indicating that high carrier concentration and high temperature are beneficial for improving carrier transport or suppressing phonon transport. Fig. 6(b) shows the temperature-dependent *ZT* of the hot-pressed Ga_1−*x*_Zn_*x*_Sb nanocomposites (*x* = 0.002, 0.005, 0.01, and 0.015). The Ga_0.99_Zn_0.01_Sb nanocomposite achieves the highest *ZT* value among all the samples. The maximum *ZT* is 0.087 at 500 K, 51% higher than the value reported for the bulk alloy in the literature.^20^ To investigate the effect of the Zn content, the *ZT* was plotted as a function of the Zn content for both 300 K and 500 K in Fig. 6(c). In Kim *et al.*'s work,^20^ the *ZT* shows a decreasing trend with the Zn content. While in our work, the *ZT* is optimized at *x* = 0.01. Either higher or lower Zn content would cause the decrease of the *ZT*. In order to enhance the *ZT* of nanocomposites, although high doping concentration should be adopted to weaken the potential barrier, there is an optimal carrier concentration since high carrier concentration will also decrease Seebeck coefficient and increase thermal conductivity.

## 4. Conclusions

In this work, we have investigated the thermoelectric properties of Zn-doped GaSb nanocomposites prepared with different ball milling conditions and amounts of Zn doping. Compared to the bulk alloy, the Seebeck coefficient of nanocomposites is increased due to the filtering effect of the potential barrier at grain boundaries, while the thermal conductivity is reduced due to the increased phonon grain boundary scattering. Both effects are beneficial for enhancing the *ZT*. While the electrical conductivity is decreased in nanograined samples because of the trapping states formed at grain boundaries. High doping concentration should be adopted to weaken the trapping effect of gain boundaries on charge carriers. On the other hand, high carrier concentration will also decrease Seebeck coefficient and increase thermal conductivity. Thus, there is an optimal carrier concentration to maximize the *ZT*. For Ga_1−*x*_Zn_*x*_Sb nanocomposites, the maximum *ZT* value is found to be 0.087 for Ga_0.99_Zn_0.01_Sb at 500 K, which is 51% higher than the value reported for the bulk alloy in the literature.^20^

## Conflicts of interest

There are no conflicts to declare.

## Supplementary Material
